# Treatment Approaches for Atypical CIDP

**DOI:** 10.3389/fneur.2021.653734

**Published:** 2021-03-15

**Authors:** Deepak Menon, Hans Dieter Katzberg, Vera Bril

**Affiliations:** Ellen & Martin Prosserman Centre for Neuromuscular Diseases, Toronto General Hospital, University Health Network, University of Toronto, Toronto, ON, Canada

**Keywords:** DADS, cidp, atypical CIDP, MADSAM, immunomodulatory therapy

## Abstract

The variants of chronic inflammatory demyelinating polyneuropathy (CIDP) differ not just in their clinical, pathological and electrophysiological characteristics, but often in their indifferent response to conventional immunosuppressive agents which are effective in typical CIDP. High quality evidence is lacking as far as the management of these atypical variants is concerned. In this review, we summarize the treatment approaches to each of these CIDP variants based on existing data. Distal acquired demyelinating symmetric polyneuropathy (DADS) has the phenotype of a symmetric, demyelinating sensory, length-dependent polyneuropathy and is frequently associated with paraproteinemia and anti myelin associated glycoprotein (MAG) antibodies. While the management of idiopathic DADS (DADS-I) is the same as CIDP, DADS-M responds suboptimally and has a favorable response to rituximab. Multifocal acquired demyelinating sensory and motor neuropathy (MADSAM) manifests as a chronic progressive demyelinating mononeuropathy multiplex which can evolve to a confluent pattern indistinguishable from CIDP. Evidence favors treating MADSAM with conventional immunomodulatory therapy (IMT), but this disorder responds less favorably than CIDP. Some patients present with purely sensory symptoms, known as pure sensory CIDP or chronic inflammatory sensory polyradiculoneuropathy (CISP), the latter localizing to a pre-ganglionic pathology. Both respond well to first line IMT, particularly to intravenous immunoglobulin (IVIG), but patients relapse without maintenance therapy. Pure motor CIDP resembles multifocal motor neuropathy with conduction block (MMNCB), but the previously reported worsening status after steroid treatment was not reproduced in recent studies, and IVIG remains the first-line therapy. Some focal forms of CIDP defy exact classification, but respond well to first-line IMT including IVIG. Overall, atypical CIDP responds to treatment with first-line IMT, but has a suboptimal response compared to CIDP. There is evidence for effectiveness with agents such as rituximab, especially in DADS-M, and this medication can also be used in cases refractory to conventional IMTs. Rituximab is also effective in CIDP with IgG4 antibodies which has distinct clinical features and is mostly refractory to first-line IMT.

## Introduction

The term “atypical CIDP” generally denotes those variants that deviate from the classical symmetrical, proximo-distal, motor, and sensory presentation of CIDP. The EFNS/PNS criteria list the following types of “atypical CIDP”: predominantly distal (distal acquired demyelinating symmetric, DADS) or asymmetric (multifocal acquired demyelinating sensory and motor neuropathy, MADSAM), focal (e.g., involvement of the brachial or lumbosacral plexus or of one or more peripheral nerves in one upper or lower limb), pure motor and pure sensory (including chronic immune sensory polyradiculopathy, CISP) (Table 1) (1). Studies have noted differences in their pathogenic mechanisms which are reflected in their atypical clinical presentations, and differences in response to conventional treatment (2, 3). CIDP itself is a rare disease with an incidence of 0.33 per 100,000 population when applying the EFNS/PNS criteria as reported in a systematic review which included studies from Europe, Australia, Japan and the United States (4). The atypical variants in a series of 376 CIDP patients constituted 18%, only a fraction of the total (5). Thus, obtaining any data for evidence-based management of CIDP variants becomes challenging. In addition, the lack of universally accepted diagnostic criteria for these entities makes matters more complicated and a second revision of the EFNS/PNS guidelines on CIDP which may include classification of these variants is anticipated. While the first-line agents for CIDP include immunomodulatory therapies (IMT), high quality evidence for their efficacy in atypical variants is lacking. In this review, we examine the current treatment approaches to each of these CIDP variants based on existing data.

**Table 1:** Summary of CIDP variants and their treatment.

**CIDP variant**	**Treatment**	**Prognosis**	**Special considerations**
DADS-I	First-line IMT	Similar to CIDP	Need for hematological evaluation and monitoring
DADS MAG	Rituximab	Less favorable for DADS M	Rare worsening with rituximab reported
MADSAM	First-line IMT Consider 2nd line agents, rituximab, in refractory cases	Generally less favorable	
Pure sensory	First-line IMT	Similar to CIDP	Responds well to IVIG or steroids
CISP	First-line IMT	Mostly similar to CIDP	Prone to relapse on tapering IMT
Pure motor	IVIG recommended as first line	Similar to CIDP	Steroid found to be equally efficacious but distinction from MMN needed
Focal variant	First-line IMT, may need maintenance therapy	Comparable to CIDP	Prone to relapse on tapering IMT
CIDP with IgG4 antibodies	Rituximab or cyclophosphamide (refractory to first-line IMT)	Poor compared to CIDP	

## Distal Acquired Demyelinating Symmetric Sensory Polyneuropathy

**Figure 1:**
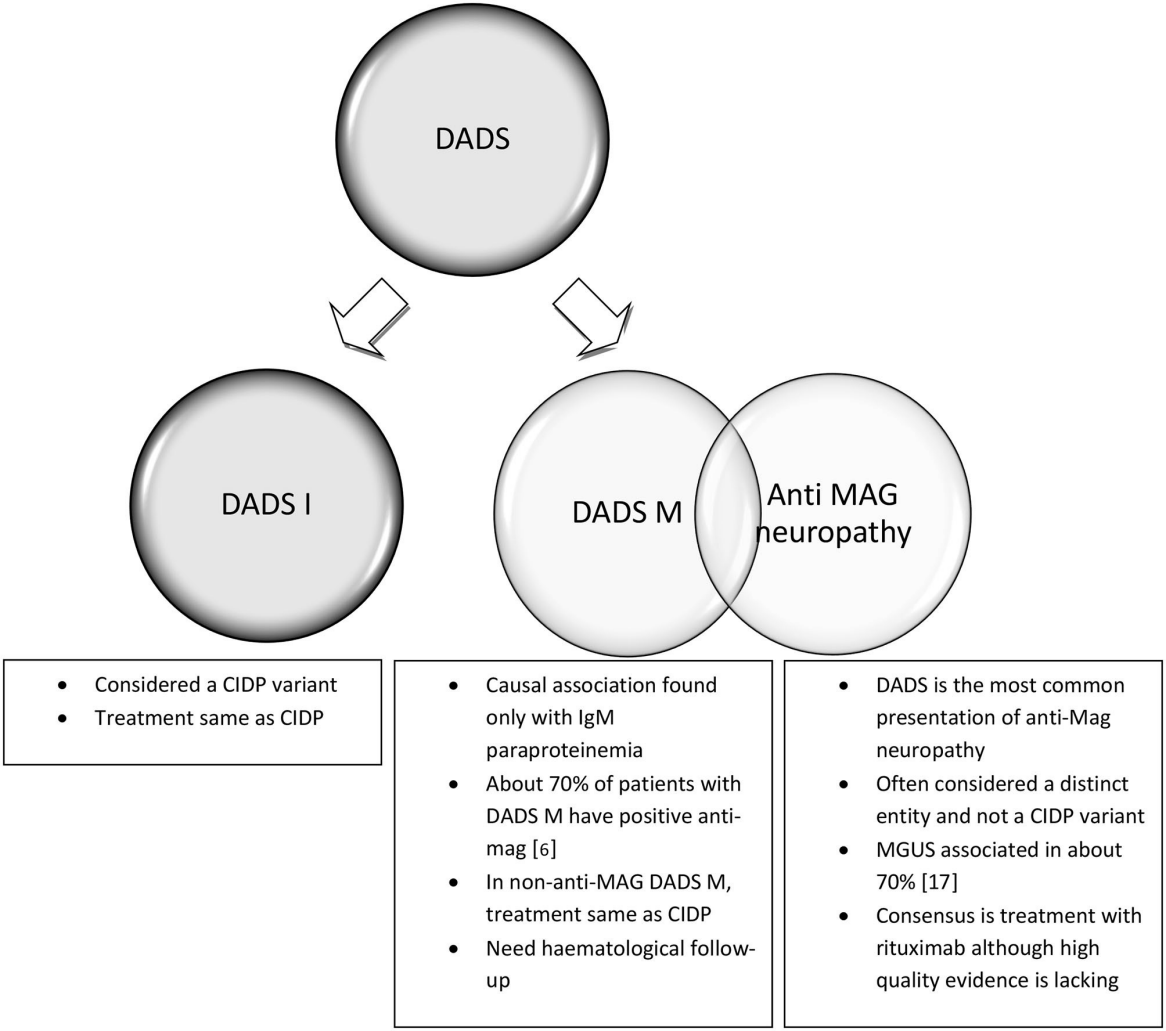
Distal acquired demyelinating sensory neuropathy (DADS) variant of CIDP and its subtypes.

Distal acquired demyelinating symmetric neuropathy (DADS) is defined by the symmetrical presentation of sensory or sensorimotor symptoms starting distally in the lower limbs without proximal limb or cranial nerve involvement and having abnormally increased distal motor latencies on nerve conduction studies (NCS) (6). DADS constitutes the most common presentation of paraprotein associated neuropathy, the others being CIDP and axonal polyneuropathy (7). IgM paraproteinemia has been most frequently associated with DADS, and in the initial description by Katz et al. (6). DADS was divided into idiopathic DADS (DADS-I) and DADS with elevated monoclonal protein (DADS-M). About 50–70% of DADS-M patients have anti-myelin associated glycoprotein (MAG) antibody comprising a discrete entity with distinctive pathology and treatment responsiveness compared to CIDP (5, 8). In fact, the presence of elevated IgM and anti-MAG are exclusionary criteria for the diagnosis of CIDP, while idiopathic DADS is considered to be a CIDP variant with similar treatment responsiveness (1, 9, 10). The overlapping clinical pictures and lack of accepted criteria, blur the distinction between paraproteinemic neuropathies, anti-MAG neuropathy, and atypical CIDP which all may present with a DADS phenotype. We will consider the treatment separately for idiopathic DADS (DADS-I), DADS with monoclonal paraproteinemia without anti-MAG antibody (DADS-M) and anti-MAG neuropathy, with or without DADS phenotype (Figure 1). Of note, the diagnosis of DADS does not mandate immunological treatment if the neuropathy is mild. In patients with no gait disability or weakness, a conservative approach with physiotherapy for balance training and periodic observation are indicated.

## Idiopathic DADS

DADS without monoclonal paraproteinemia or anti-MAG antibody, also referred to as DADS-I, is considered as a variant of CIDP. The initial report by Katz et al. (6) demonstrated that DADS-I resembled CIDP in having a similar 70–80% response rate to conventional treatment including intravenous immunoglobulin (IVIG), plasma exchange (PLEX), and prednisone. In contrast, data from larger cohorts indicated that treatment response with IMT was significantly lower for DADS compared to CIDP (64 vs. 87%, respectively), but a distinction was not drawn between different DADS subtypes (5). Specific data on treatment response of DADS without MAG is limited to a single study which found that that the majority of patients responded to conventional IMT (9). This study included 10 patients, and 9 had underlying hematological conditions such as chronic lymphocytic leukemia (CLL), Hodgkin's lymphoma or monoclonal gammopathy of unknown significance (MGUS) (9). With the existing evidence, there is no reason to treat patients having DADS without MAG differently than those with typical CIDP although the treatment response may be less robust. It is essential to perform a thorough evaluation to discover any co-existing hematological conditions.

## DADS-M Without Anti-Mag Antibodies

A causal association of monoclonal gammopathy with DADS is significantly stronger for IgM than for IgG or IgA monoclonal gammopathies (11). From the neuropathy perspective, treatment is based on the severity, progression, and motor deficits rather than the level of M protein (7). In the initial report by Katz et al., the prognosis of DADS-M was less favorable compared to DAD I or CIDP, but this cohort did not distinguish patients with anti-MAG antibodies and the latter respond less favorably to treatment (6, 8). In a small series of patients without anti-MAG DADS, the response to treatment with IMT was similar to CIDP although nine out of the 10 patients had associated hematological conditions such as MGUS and CLL (9). From a hematological standpoint, a definitive treatment may be required depending on the condition. In cases such as MGUS or asymptomatic Waldenstrom's macroglobulinemia, regular follow-up with serum protein electrophoresis (SPEP) and serum immunoelectrophoresis (SIEP) should be undertaken given the 1% chance per year of malignant transformation (11–13). In addition to these laboratory parameters, clinical features such as anemia, bone pain, lymphadenopathy, hepatosplenomegaly, or hyperviscosity symptoms suggest a transition to multiple myeloma or Waldenstroms macroglobulinemia (14, 15). Limited evidence suggests a favorable response to first-line IMT for non-MAG DADS neuropathy (9).

## Anti-Mag Neuropathy With OR Without DADS Phenotype

The pathology of anti-MAG positive DADS, involves deposits of IgM and complement, with splitting of myelin lamellae resulting in demyelination and conduction block. The response to treatment in DADS with paraproteinemia (DADS-M) to the first line agents effective in CIDP is suboptimal (1, 8). Initial reports demonstrated a 30% response rate with minimal subjective improvement in patients with DADS-M while patients with DADS-I or CIDP had nearly 70–95% objective improvement with conventional IMT (6). About 70% of patients with DADS-M in this cohort had anti-MAG antibody positivity, confounding the results as those with anti-MAG antibody do not respond well to first-line CIDP treatments. Most authors consider anti-MAG neuropathy distinct from CIDP due to the pathological differences (16, 17). Uncontrolled studies and randomized placebo-controlled trials provided some evidence regarding the benefits of rituximab for anti-MAG DADS neuropathy (5, 18–20). Factors such as INCAT disability scores and time to walk 10 m improved with rituximab treatment (19, 20). However, the INCAT sensory sum score which reflects the sensory deficits, did not show improvement creating doubts that this scale is the ideal outcome measure to use in the DADS phenotype (21). Chemotherapeutic agents such as cyclophosphamide, fludaribine, and chlorambucil have been explored, either as monotherapy, or in combination with first line IMT. However, the potential risk of future malignancies and their side-effect profiles make their use limited except in refractory cases (3, 5, 12, 13). A recent Cochrane review concluded that (a) good quality evidence was lacking to recommend any immunotherapy in anti-MAG neuropathies and (b) IVIG and rituximab may not offer clinically significant benefit in anti-MAG neuropathy, given the low quality of evidence (22). There have been isolated reports of patients worsening with rituximab that also need to be considered during patient counseling and monitoring (23). Despite these concerns, most authorities recommend rituximab as the treatment of choice in patients with disabling anti-MAG neuropathy (12, 21).

## MADSAM

MADSAM is a painless, demyelinating, mononeuropathy multiplex, and is the most frequently encountered variant of CIDP in most series. The distinct nature of MADSAM pathology is that the brunt of the macrophage-mediated demyelination is multifocal and distributed mainly in mid-limb or proximal nerve segments (24, 25). In their seminal paper on MADSAM, Saperstein et al. noted a treatment response rate of 56% with IVIG and 50% for prednisone, with other studies reporting an overall response rate of 70% (26, 27). The responses in different muscle groups varied from marked to none, and the benefit lasted for several months after treatment discontinuation (26). Further studies have shown that in MADSAM the (a) treatment responses to first line agents (b) long term outcomes and (c) rates of remission are inferior compared to typical CIDP. The response rates to IVIG, PLEX, and prednisone have varied in different reports, but overall these treatments were similarly effective (2, 5, 27, 28). About 25% of patients refractory to first-line therapies were subsequently treated with cyclophosphamide and azathioprine with discouraging results (2, 27). Although there are anecdotal reports of patients with MADSAM responding to rituximab after failing to respond to IVIG, PLEX, corticosteroids, and mycophenolate, prospective data regarding second-line treatment options are not available (29). The current evidence thus suggests conventional IMT as first-line therapy in patients with MADSAM followed by chemotherapeutic agents or rituximab as second-line agents in refractory cases.

## Pure Sensory CIDP

The pure sensory variant of CIDP was initially recognized by Oh et al. (30) who first described a patient presenting with a progressive pure sensory neuropathy with demyelinating features affecting sensory and motor peripheral nerves on NCS. In their initial report, patients were noted to be steroid responsive and only in a minority were PLEX and other steroid sparing agents required. IVIG was not employed in this initial series. Subsequent larger studies have noted no difference in treatment response in comparison with typical CIDP, with IVIG and steroids being equally efficacious (5). About 90% of patients are reported to respond to IVIG or steroids in most series with only very few patients requiring PLEX or alternate immunological agents (5, 31–33). There are rare reports of a patient deteriorating with one of the first-line agents but then responding to another agent, such as rituximab (34–36). In general, the treatment and expected response of pure sensory CIDP are similar to typical CIDP.

## Chronic Inflammatory Sensory Polyneuropathy

CISP is often considered a pure sensory CIDP due to its similarities in clinical presentation, but with the distinctive feature of sensory pre-ganglionic root involvement as evidenced by normal sensory NCS, abnormal sensory evoked potentials (SEP), and thickened spinal roots on MRI. In the initial case series of 15 patients with CISP, six patients were significantly disabled and required treatment, four of whom received IVIG and two received steroids (37). All of the treated patients had a rapid improvement, but relapsed on attempted tapering. Regional variants affecting one limb with motor symptoms, reminiscent of focal CIDP are also reported, but are distinct from the latter in having normal sensory nerve conduction study parameters. Such cases defy exact classification but such patients have responded well to IVIG (38).

## Pure Motor CIDP

Pure motor CIDP resembles multifocal motor neuropathy (MMN) in its clinical presentation but is more symmetrical and is classified as an atypical form of CIDP. Motor conduction blocks are the most common electrophysiological finding in this entity, and in many cases there is an absence of sensory nerve conduction abnormalities, again similar to MMN (5, 35). From a clinical standpoint, this entity also resembles motor neuron disease (MND) and can create diagnostic confusion, but the absence of any bulbar involvement and the presence of demyelinating features on NCS are helpful in distinguishing pure motor CIDP from MND (39). Some of the initial case series of this entity reported unresponsiveness or worsening with steroids while having an excellent response to IVIG (40–42). These early reports led to the EFNS/PNS guidelines of 2010 recommending IVIG as the initial treatment in pure motor CIDP. Given the challenges in distinguishing motor CIDP and MMN, some of these early studies may have included patients with MMN which typically is steroid resistant and IVIG responsive (43). Several subsequent case series have failed to substantiate steroid resistance in pure motor CIDP. Data from the Italian data base revealed a steroid response rate of 43% in pure motor CIDP in comparison with 51% for typical CIDP. Other studies have shown a response rate of 80% with steroids, and 75% with IVIG (44). More importantly, patients treated with steroids did not have any worsening in either of these studies. The overall treatment response rate to IMT has been 70–90% in most series and is comparable to CIDP. Given the fact that a clear distinction is often difficult to establish between pure motor CIDP and MMN, IVIG may still be the ideal initial choice, if all other factors are equal. The upcoming revision of EFNS/PNS guidelines on CIDP may better define how to distinguish pure motor CIDP from MMN and clarify the use of steroids and IVIG in its treatment.

## Focal CIDP

Focal CIDP remains the least defined of CIDP variants and the least frequent. Focal CIDP was not seen in two large cohorts of patients with CIDP variants from Italy (*n* = 84) and Japan (*n* = 40) (2, 5). The initial reports of a monomelic demyelinating polyneuropathy with hypertrophy of the involved nerves and biopsy showing characteristic “onion bulb” changes led to recognition of this focal form of CIDP (38). It was recognized to be distinct from MMN in having sensory involvement, absence of anti-GM1 ganglioside antibodies and a favorable response to steroids. In one of the earliest case series by Thomas et al. (45), all but one patient responded satisfactorily to either IVIG or steroid treatment, but required long term maintenance treatment due to relapse on attempted tapering. It is possible that the lack of uniformity in nomenclature and the absence of well-accepted criteria has led to the under reporting of focal CIDP. Some of the reports in the literature of inflammatory plexitis and inflammatory mononeuropathies might be re-classified as focal CIDP, and these patients may respond well to IVIG or steroids (46–48). In addition, focal CIDP may be considered at one end of a spectrum of disease, as an arrested form of MADSAM or CIDP, and thus would respond to similar treatment strategies (49). Focal CIDP seems to be responsive to IVIG or steroids with requirement for long term maintenance treatment in many patients due to higher chances of relapse with attempted tapering.

## CIDP With IgG4 Antibodies

Investigations for possible biomarkers of CIDP have led to the identification of pathogenic autoantibodies directed against several nodal and paranodal antigens amongst the subset of patients with CIDP (50). IgG4 antibodies directed against some of these paranodal antigens, namely neurofascin (Nfasc 155 and Nfasc 140/186), contactin-1 (CNTN1), and contactin associated protein-1 (Caspr1), result in a demyelinating polyneuropathy resembling CIDP but with distinct clinical features such as early age of onset, subacute presentation, presence of tremor and ataxia, and poor responsiveness to first-line agents (51, 52). Only a small number of patients with refractory CIPD have one of these autoantibodies. In refractory CIDP, including those patients with IgG4 antibodies, treatment with cyclophosphamide or rituximab has resulted in a favorable response although the evidence is restricted to relatively small case series (50–53). With the demonstrated efficacy and safety profile in several autoimmune disorders, current evidence favors the use of rituximab in these patients. The results of a randomized double-blind placebo-controlled trial on the efficacy and safety of rituximab in refractory CIDP patients, with or without IgG4 antibodies, should provide better evidence in this patient population (54).

## Conclusion

The data on the treatment of atypical variants of CIDP are of low-quality and limited in patient numbers. Large prospective series or clinical trials are non-existent other than possibly for the DADS phenotype. Furthermore, the diagnostic separation of these variants is not clearly demarcated and some entities such as DADS or pure motor CIDP and focal CIDP may represent overlapping phenotypes. These variants can be impossible to distinguish from other entities such as MMN or MADSAM. Other than in anti-MAG positive DADS, the existing literature indicates a favorable treatment response with conventional first-line IMT used in typical CIDP. Given reports of some patients with pure motor and pure sensory CIDP worsening with steroids, IVIG may be preferred as the first-line agent in these variants. Agents such as rituximab are currently being re-explored in anti-MAG neuropathy and in IgG4 antibody associated CIDP earlier in the treatment algorithm. A revision of the guidelines to clearly define the diagnostic criteria for these entities is required in order to undertake prospective clinical trials and improve our understanding of atypical CIDP.

## Author Contributions

DM was involved in the review of literature, drafting the work, and revising it critically for important intellectual content. HK was involved in drafting the work and revising it critically for important intellectual content. VB was involved in designing, review of literature, revising it critically for important intellectual content, and final approval of the version. All authors contributed to the article and approved the submitted version.

## Conflict of Interest

The authors declare that the research was conducted in the absence of any commercial or financial relationships that could be construed as a potential conflict of interest.
